# Assessment of novel vaccination regimens using viral vectored liver stage malaria vaccines encoding ME-TRAP

**DOI:** 10.1038/s41598-018-21630-4

**Published:** 2018-02-21

**Authors:** Carly M. Bliss, Georgina Bowyer, Nicholas A. Anagnostou, Tom Havelock, Claudia M. Snudden, Huw Davies, Simone C. de Cassan, Amy Grobbelaar, Alison M. Lawrie, Navin Venkatraman, Ian D. Poulton, Rachel Roberts, Pooja B. Mange, Prateek Choudhary, Saul N. Faust, Stefano Colloca, Sarah C. Gilbert, Alfredo Nicosia, Adrian V. S. Hill, Katie J. Ewer

**Affiliations:** 1 The Jenner Institute, University of Oxford, Oxford, UK; 2 NIHR Wellcome Trust Clinical Research Facility, University of Southampton, University Hospital Southampton NHS Foundation Trust, Southampton, UK; 3 Department of Medicine, Division of Infectious Diseases, University of California, Irvine, CA USA; 4 ReiThera (formerly Okairos), 00144 Rome, Italy; 5 CEINGE, Via Comunale Margherita, 484-538, 80131 Napoli, Italy; 6 Department of Molecular Medicine and Medical Biotechnology, University of Naples Federico II, Naples, Italy

## Abstract

Heterologous prime-boost vaccination with viral vectors simian adenovirus 63 (ChAd63) and Modified Vaccinia Ankara (MVA) induces potent T cell and antibody responses in humans. The 8-week regimen demonstrates significant efficacy against malaria when expressing the pre-erythrocytic malaria antigen Thrombospondin-Related Adhesion Protein fused to a multiple epitope string (ME-TRAP). We tested these vaccines in 7 new 4- and 8- week interval schedules to evaluate safety and immunogenicity of multiple ChAd63 ME-TRAP priming vaccinations (denoted A), multiple MVA ME-TRAP boosts (denoted M) and alternating vectors. All regimens exhibited acceptable reactogenicity and CD8^+^ T cell immunogenicity was enhanced with a 4-week interval (AM) and with incorporation of additional ChAd63 ME-TRAP vaccination at 4- or 8-weeks (AAM or A_A_M). Induction of TRAP antibodies was comparable between schedules. T cell immunity against the ChAd63 hexon did not affect T cell responses to the vaccine insert, however pre-vaccination ChAd63-specific T cells correlated with reduced TRAP antibodies. Vaccine-induced antibodies against MVA did not affect TRAP antibody induction, and correlated positively with ME-TRAP-specific T cells. This study identifies potentially more effective immunisation regimens to assess in Phase IIa trials and demonstrates a degree of flexibility with the timing of vectored vaccine administration, aiding incorporation into existing vaccination programmes.

## Introduction

Malaria is a major global health problem, with more than 200 million cases per year resulting in over 400,000 deaths, predominantly in sub-Saharan Africa. A further 3.4 billion people are estimated to be at risk of contracting the disease^1^. Development of a prophylactic vaccine is considered necessary for disease eradication^2^, with vaccination proving successful in elimination programmes against smallpox^3^ and polio^4^ through induction of antibodies. However, successful vaccination against malaria is likely to benefit from induction of both cellular and humoral immunity^5–7^, with the cellular arm providing cytotoxic cell-mediated protection against the pre-erythrocytic liver stage of the disease, in addition to CD4^+^ T cell help and B cell antibody induction against the sporozoite and blood stages of infection^8^.

Adenovirus vectors induce high levels of both T cells and antibodies, particularly CD8^+^ effector and effector memory responses^9,10^. The chimpanzee adenovirus serotype 63-derived viral vector (ChAd63) has been widely evaluated in humans for malaria and is safe and highly immunogenic^10–12^. Seroprevalence of neutralising antibodies to ChAd63 is lower than to human adenovirus vectors, such as AdHu5, in many populations including African children^13,14^.

Following vaccination with Modified Vaccinia Ankara (MVA) virus, infected antigen-presenting cells rapidly migrate to draining lymph nodes presenting antigen to naïve CD8^+^ T cells as rapidly as 6 hours after inoculation^15^. Thus poxviruses are able to stimulate the cytotoxic arm of the adaptive immune response and induce accelerated development of CD8^+^ T cell memory^16^, but are particularly effective at boosting pre-primed T cell and antibody responses.

ChAd63 and MVA viral vectors encoding the pre-erythrocytic Thrombospondin-Related Adhesion Protein (TRAP) antigen^17^, fused to a string of multiple CD4^+^ and CD8^+^ malaria epitopes (ME)^18,19^, have been tested in many studies using a standard 8 week prime-boost regime: ChAd63.MVA ME-TRAP. In Phase I and II clinical vaccine trials, this candidate vaccination regimen induced high numbers of antigen-specific T-cells in humans, and upon controlled malaria infection (CHMI) of malaria naïve adults, demonstrated 21% sterile efficacy and a delay to parasitaemia in a further 36% of vaccinees^20^. The 8-week regimen induced a peak median *ex vivo* IFN-γ ELISpot response of over 2400 SFC/10^6^ PBMC. Protective efficacy positively correlated with the frequency of monofunctional CD8^+^ IFN-γ^+^ T cells measured one day prior to challenge (C-1) and CD8^+^CD107a^+^ T cells 150 days post challenge (C + 150). Furthermore, a 67% reduction in malaria infection was measured in ChAd63.MVA ME-TRAP vaccinated adults in a malaria endemic region of coastal Kenya. In this setting, IFN-γ-secreting T cell responses measured in an *ex vivo* ELISpot assay, directed towards the mid-section of the TRAP antigen correlated with protection^21^.

It has been previously demonstrated in mice that the phenotype of T cell memory may differ depending on number of vaccinations. In the absence of anti-vector responses, secondary and tertiary responses to vaccination may result in the generation of memory CD8^+^ T cells that retain effector-like properties as opposed to central memory T cell phenotypes, and preferentially accumulate in non-lymphoid tissues^22^. Furthermore, in mice primed with adenovirus and boosted with MVA both encoding ME-TRAP, blood CD8^+^ T cells of an effector memory phenotype correlate with protection against malaria liver-stage infection following *Plasmodium berghei* challenge^16^.

Flexibility in the 8-week prime-boost interval of ChAd63.MVA ME-TRAP is currently untested in human vaccinees. In the murine challenge model, the chimpanzee adenovirus 9 (AdC9) encoding ME-TRAP and MVA ME-TRAP were assessed alone and in prime-boost combination by sporozoite challenge at 2 and 8 weeks post boost. The highest degree of sterile protection was achieved when mice were primed with AdC9 ME-TRAP and boosted with MVA ME-TRAP with an 8-week interval (96% when challenged at 2 weeks post boost and 59% when challenged at 8 weeks post boost), when compared to MVA.ChAd9, MVA alone and ChAd9 alone. Shorter prime boost intervals of 1, 2 and 4 week intervals using this regimen were also efficacious^16^.

The use of multiple homologous ChAd63 ME-TRAP vaccinations has not previously been tested in humans and it is unknown whether multiple administrations would enhance regimen immunogenicity or impair it by boosting pre-existing anti-vector immunity. Preliminary data, whereby a small number of human subjects administered the ChAd63.MVA ME-TRAP regimen were re-boosted with either ChAd63 ME-TRAP or MVA ME-TRAP after a long interval averaging 8 months, show that T cell responses can indeed be re-boosted by both a delayed MVA administration (A_M__M) and a delayed ChAd63 administration (A_M__A)^10^.

The first Phase I clinical vaccine study of ChAd63.MVA ME-TRAP saw > 90% of vaccinees seroconvert to the ChAd63 vector following vaccination, but found no correlation between baseline ChAd63 antibody titres and peak ELISpot response to the vaccine insert post MVA ME-TRAP boost^10^. Subsequent trials have identified a trend towards higher peak T cell responses to ME-TRAP in volunteers with pre-existing anti-vector antibodies^20^, and a negative correlation between pre-vaccination ChAd63 titre and post-ChAd63 T cell response to TRAP^23^. In a hepatitis C (HCV) clinical vaccine trial, a positive correlation was measured between boosting of adenovirus-specific T cells and that of the HCV-specific T cell response, following administration of AdC3 or AdHu6 encoding non-structural HCV proteins^11^.

Data from macaques indicates that vaccination schedules containing more than one administration of an adenoviral-vectored vaccine component generate enhanced cell -mediated immunogenicity^24^. A similar macaque study found that boosting with MVA ME-TRAP after a ChAd63 ME-TRAP prime, increased both the magnitude and breadth of the immune response to the vaccine insert^25^, however subsequent MVA boosts did not broaden the responses compared with a single MVA boost^25^. After MVA vaccination, responses measured by *ex vivo* ELISpot peaked and then declined to around 30% of the peak level. Further MVA ME-TRAP vaccinations boosted the magnitude of the T cell and antibody responses, however the peak frequencies obtained after the second and third MVA immunisations did not surpass levels achieved by a single MVA ME-TRAP administration.

Vaccine-induced T cells contribute towards protection against malaria following vaccination with the ChAd63.MVA ME-TRAP regimen^20,21^. Greater vaccine efficacy might be achieved by inducing higher frequencies of antigen-specific T cells through administration of ChAd63 ME-TRAP and MVA ME-TRAP via already established doses and routes, but according to novel schedules (summarised in Table 1).

**Table 1:** Group vaccine regimens and schedule abbreviation.

Group (n = 6)	Time Point of Vaccine Administration						Schedule Abbreviation
	Day 0	4 weeks	8 weeks	12 weeks	16 weeks	24 weeks	
1	ChAd63 ME-TRAP	ChAd63 ME-TRAP	ChAd63 ME-TRAP		MVA ME-TRAP		AAA_M
2	ChAd63 ME-TRAP		ChAd63 ME-TRAP		MVA ME-TRAP	MVA ME-TRAP	A_A_M_M
3	ChAd63 ME-TRAP	ChAd63 ME-TRAP	MVA ME-TRAP	MVA ME-TRAP			AAMM
4	ChAd63 ME-TRAP		MVA ME-TRAP		MVA ME-TRAP	MVA ME-TRAP	A_M_M_M
5	ChAd63 ME-TRAP	MVA ME-TRAP	ChAd63 ME-TRAP		MVA ME-TRAP		AMA_M
6	ChAd63 ME-TRAP	MVA ME-TRAP	ChAd63 ME-TRAP	MVA ME-TRAP			AMAM
7	ChAd63 ME-TRAP		MVA ME-TRAP		ChAd63 ME-TRAP	MVA ME-TRAP	A_M_A_M
Standard Regimen (G4 + 7)	ChAd63 ME-TRAP		MVA ME-TRAP				A_M
Shortened Regimen (G5 + 6)	ChAd63 ME-TRAP	MVA ME-TRAP					AM

“A” denotes ChAd63 ME-TRAP, “M” denotes MVA ME-TRAP and “_” denotes an 8-week interval, no underscore denotes a 4 -week interval. See supplementary Figure S2 for CONSORT diagram.

## Results

### Safety

A CONSORT diagram showing volunteer enrolment, exclusion, allocation and follow up is shown in Figure S2. There were no serious adverse events related to ChAd63 ME-TRAP 5 × 10^10^ v.p. vaccination or MVA ME-TRAP 2 × 10^8^ p.f.u. vaccination. Adverse events (AE) were generally mild in nature and over 90% resolved spontaneously or with simple analgesia within 48 hours of onset. As seen in previous studies of these vaccines, ChAd63 ME-TRAP vaccination was less reactogenic than MVA ME-TRAP vaccination, with 4 volunteers developing severe AEs compared to 10, respectively. Two of the volunteers experiencing severe AEs after ChAd63 had 2 separate severe AEs each, all were classified as possibly related to vaccination (detailed in Table S1a). Only one severe AE after ChAd63 immunisation was judged to be definitely related to vaccination and consisted of vaccine site swelling, which resolved within 48 hours. Six of the volunteers reporting AEs after MVA had more than one separate severe AE and 8/18 (44%) were judged as definitely related to vaccination. One-third (6/18) of severe AEs after MVA vaccination resolved within 48 hours (Table S1b). There were no significant differences in local and systemic reactogenicity when comparing double and triple ChAd63 ME-TRAP priming between groups 1, 2 and 3. Repeated MVA ME-TRAP boosts in group 4 elicited reactogenicity profiles that were not significantly different to the initial MVA ME-TRAP boost. There was no increase in AEs with either vaccine when they were alternately administered twice in groups 5, 6 and 7. The number of local and systemic AEs after each vaccination in each group are summarised in Table S2.

### Cellular immunogenicity measured by *ex vivo* IFN-γ ELISpot

**Figure 1:**
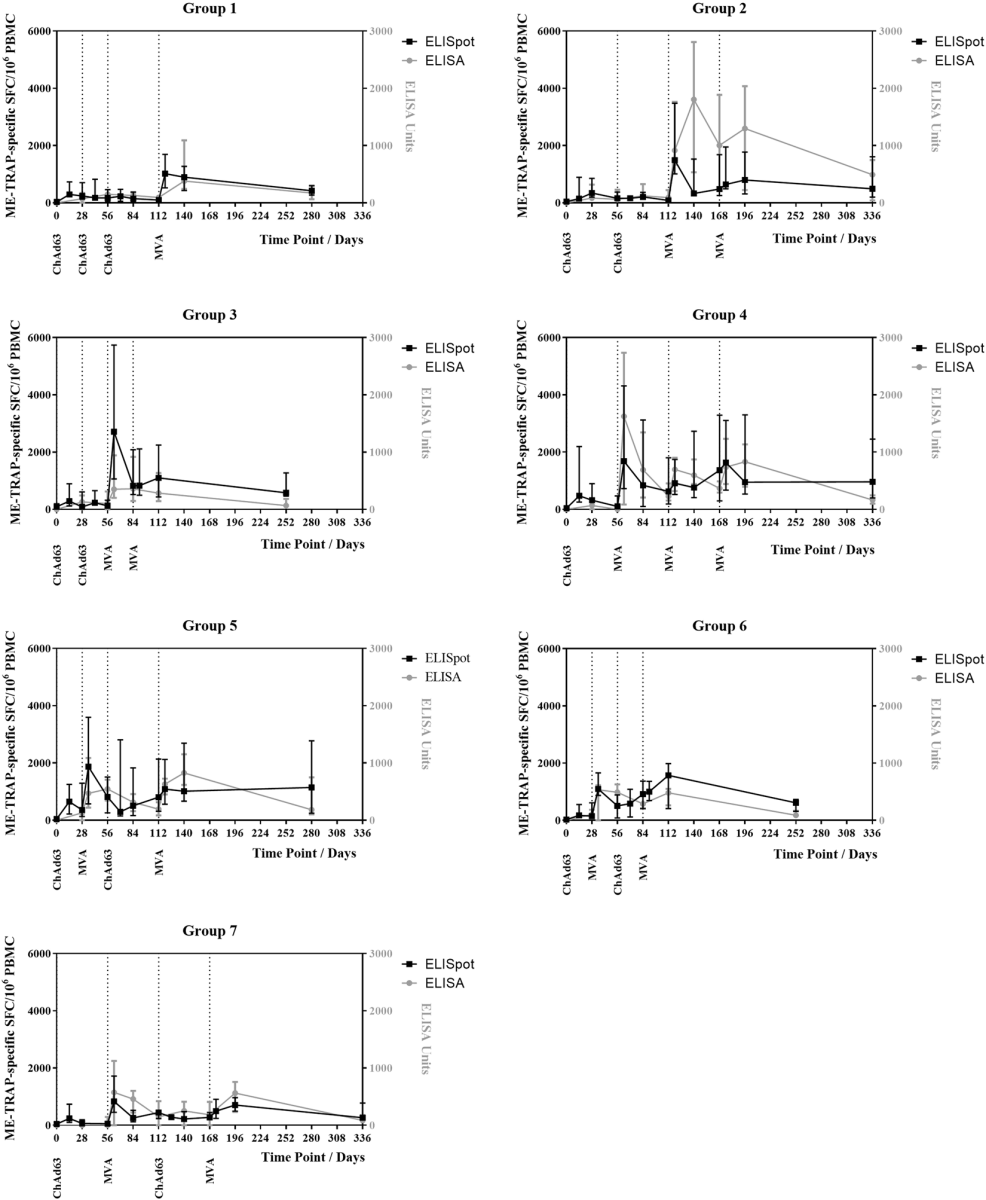
T cell and antibody response kinetics. **(A**–**G**) Time course of ME-TRAP T cell response by *ex vivo* IFN-γ ELISpot and TRAP antibody response by ELISA, in groups 1–7 respectively. Median with interquartile range displayed.

**Figure 2:**
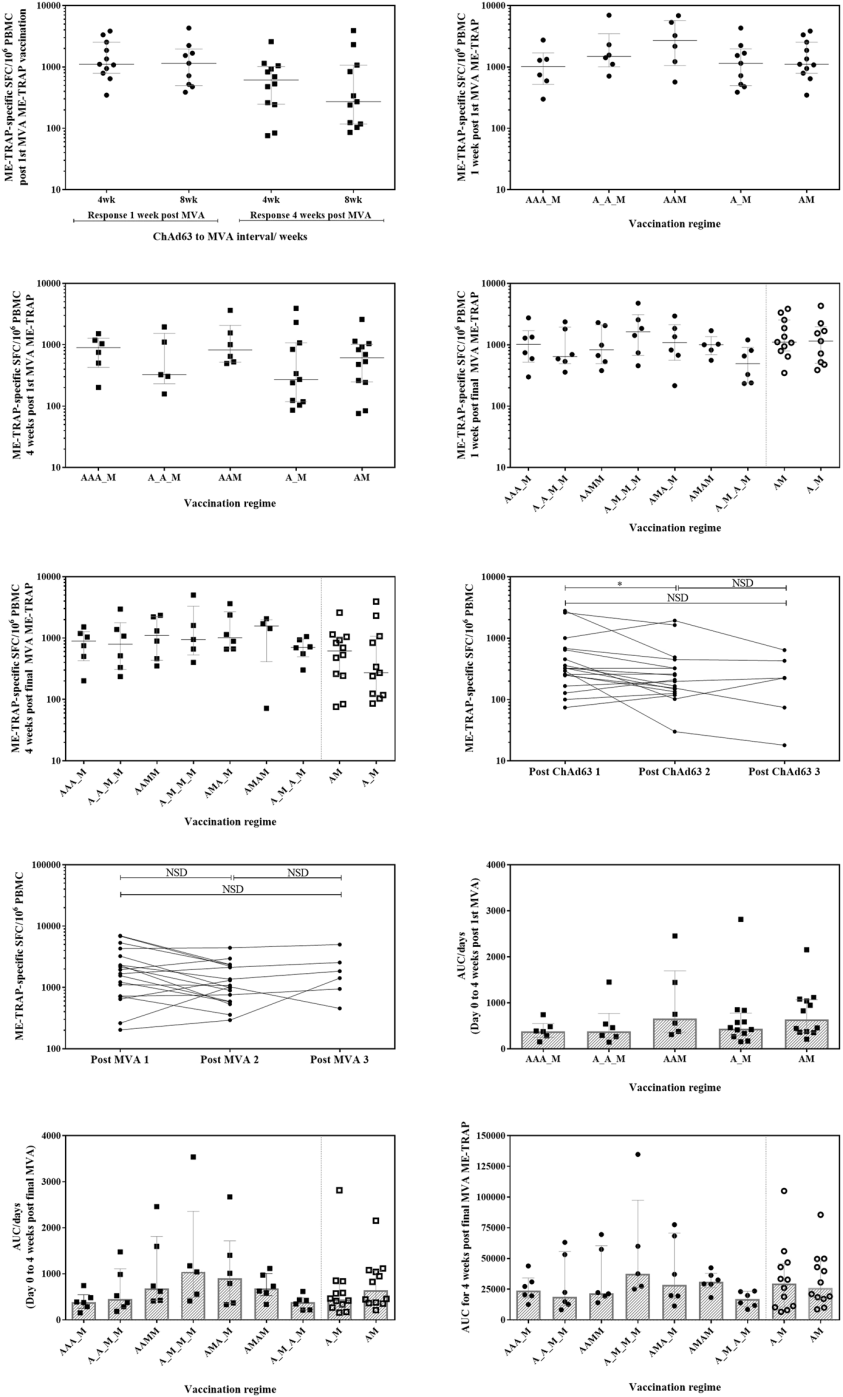
*Ex vivo* IFN-γ ELISpot response. (**A**) 1 week and 4 weeks post-MVA ME-TRAP responses for volunteers boosted at 4 weeks compared to 8 weeks post ChAd63 ME-TRAP. No significant difference at 1 or 4 weeks post boost, Mann Whitney test p = 0.7103 and p = 0.6947, respectively. (**B**) 1 week and (**C**) 4 weeks post-MVA ME-TRAP response following single, double and triple ChAd63 ME-TRAP priming vaccination(s), with single MVA ME-TRAP boost. No significant difference between groups by Kruskal-Wallis ANOVA between all groups with Dunn’s correction p = 0.3722 and p = 0.5233, respectively. (**D**) 1 week and (**E**) 4 weeks post final MVA ME-TRAP vaccination in each group, with prime-boost regimens of 4 and 8 weeks displayed for reference. No significant difference between groups by Kruskal-Wallis ANOVA between all groups with Dunn’s correction p = 0.4284 and p = 0.4519, respectively. (**F**) Peak *ex vivo* IFN-γ ELISpot response following each ChAd63 ME-TRAP response per volunteer. Wilcoxon matched pairs signed rank test ChAd63 ME-TRAP 1 and ChAd63 ME-TRAP 2, p = 0.0305. (**G**) Peak *ex vivo* IFN-γ ELISpot response following each MVA ME-TRAP response per volunteer. (**H**) Individual AUC response for day 0 to 4 weeks post 1st MVA ME-TRAP vaccination. No significant difference between groups by Kruskal-Wallis ANOVA with Dunn’s correction p = 0.3675. (**I**) Individual AUC response for day 0 to 4 weeks post final MVA ME-TRAP vaccination. No significant difference between the 4-dose or 2 -dose regimens. Kruskal-Wallis ANOVA with Dunn’s correction for multiple comparisons between groups, p = 0.1347. (**J**) Individual AUC response for day of final MVA ME-TRAP boost to 4 weeks later. No significant difference between groups by Kruskal-Wallis ANOVA with Dunn’s correction between groups, p = 0.2042. Median with IQR displayed. A-G) Circles and squares denote responses 1 week and 4 weeks post vaccination, respectively.

After immunisation with ChAd63 ME-TRAP, 96% of vaccinees had a positive response to vaccination, as measured by *ex vivo* IFN-γ ELISpot (Fig. 1, individual data points not displayed). Of the 2 initial non-responders, both responded positively after either a second ChAd63 ME-TRAP vaccination at day 56 (group 2), or MVA ME-TRAP vaccination at day 28 (group 5). Median time courses of *ex vivo* ELISpot response by group are shown in Fig. 1. Direct comparison of peak immunogenicity was used to assess the effect of a 4- or 8-week prime-boost interval with ChAd63 ME-TRAP priming and MVA ME-TRAP boosting vaccinations. ELISpot responses for the 12 volunteers boosted after a 4-week (group 5, 6) compared with an 8-week interval (group 4, 7) post ChAd63 ME-TRAP vaccination were not significantly different at 1 or 4 weeks post MVA ME-TRAP vaccination (Fig. 2A, Mann Whitney test p = 0.71 and p = 0.69, respectively).

The administration of 2 or 3 ChAd63 ME-TRAP vaccinations did not enhance the post-MVA ME-TRAP ELISpot response at 1 or 4 weeks post first MVA ME-TRAP vaccination (Fig. 2B,C Kruskal-Wallis ANOVA with Dunn’s correction p = 0.52 and p = 0.37 respectively) or the response measured 1 and 4 weeks post MVA ME-TRAP vaccination (Fig. 2D,E, Kruskal-Wallis ANOVA with Dunn’s correction, p = 0.43 and p = 0.45 respectively).

The kinetics of ME-TRAP-specific responses were similar across all groups following ChAd63 ME-TRAP prime, peaking 14–28 days post-vaccination. The ELISpot response after the first ChAd63 ME-TRAP vaccination was significantly higher than after the second vaccination (Fig. 2F, Wilcoxon matched pairs signed rank test for individual volunteer peak response, p = 0.03) and showed a downward trend after the third vaccination (Fig. 2F, Wilcoxon matched pairs signed rank test for individual volunteer peak response, p = 0.063). In groups with multiple MVA ME-TRAP vaccinations, the peak response to the first MVA ME-TRAP was at 7 days, but following a second MVA, responses peaked after 28 days or later (data not shown). The highest peak in ELISpot response was measured after the first dose of MVA ME-TRAP in 61% of volunteers. Paired analysis of individual volunteer peak responses after consecutive MVA ME-TRAP vaccination in group 2 (A_A_M_M), group 3 (AAMM) and group 4 (A_M_M_M) showed no significant boosting in peak response with subsequent MVA ME-TRAP vaccinations (Fig. 2G, Wilcoxon matched-pairs signed rank test between MVA ME-TRAP 1 and MVA ME-TRAP 2 p = 0.07, MVA ME-TRAP 1 and MVA ME-TRAP 3 p = 0.16 or MVA ME-TRAP 2 and MVA ME-TRAP 3 p = 0.31).

We used an area-under-curve (AUC) analysis to undertake a more comprehensive analysis of vaccine-induced immunogenicity. The AUC value is calculated per volunteer and represented as a proportion of the regimen length (AUC/days) from day 0 to 4 weeks post first or final MVA ME-TRAP vaccination. It is representative of both the peak IFN-γ ELISPOT response to ME-TRAP and maintenance of this response over the defined time period. This scaled AUC analysis showed no differences in immunogenicity with the different priming schedules up to 4 weeks after the first MVA ME-TRAP dose (Fig. 2H Kruskal-Wallis ANOVA with Dunn’s correction, p = 0.37), and demonstrated no improvement of any 4-dose vaccination regimen over the 8-week 2 -dose standard ChAd63.MVA ME-TRAP prime-boost regimen. Although no significant differences between groups were detected by this analysis, group 4 (A_M_M_M) consistently demonstrated the largest responses by AUC, with the response maintained by subsequent MVA ME-TRAP boosts (Fig. 2I).

To specifically evaluate response durability, AUC was calculated for all groups from the point of final MVA ME-TRAP vaccination for a fixed follow up period of 4 weeks. Durability was comparable between all groups (Fig. 2J Kruskal-Wallis ANOVA with Dunn’s correction between all groups, p = 0.5099).

**Figure 3:**
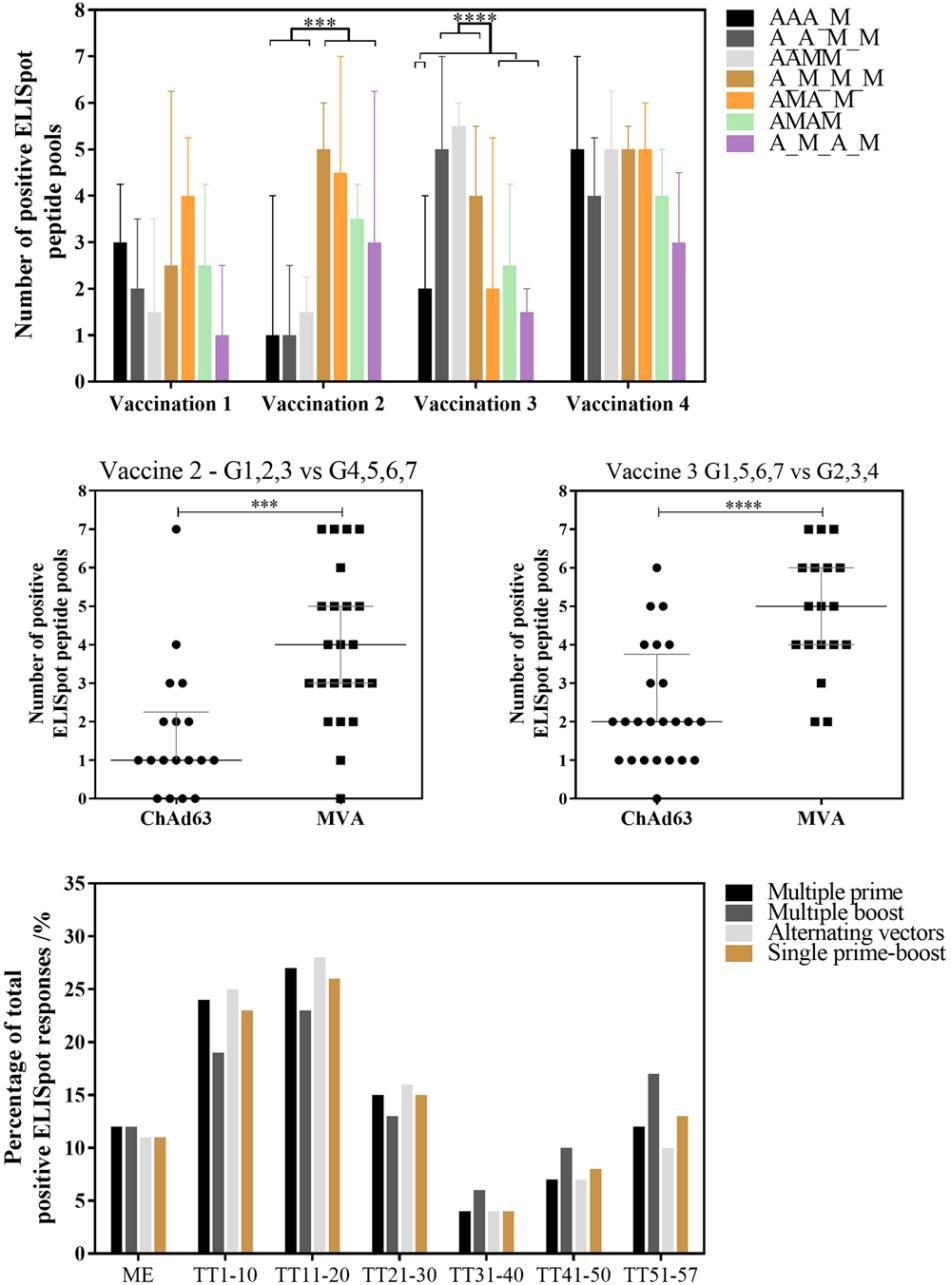
Breadth of T cell response. (**A**) Response breadth by number of positive ELISpot pools per group at the peak post vaccination: 14–28 days post-ChAd63 ME-TRAP and 7–28 days post-MVA ME-TRAP. Median plus interquartile range displayed to a maximum of 7 pools: TRAP T9/96 (TT) pool 1–6 and multiple epitope pool. Response breadth to ChAd63 ME-TRAP and MVA ME-TRAP by Mann-Whitney test at vaccination 2 and 3, p = 0.0002 and p < 0.0001 respectively, as shown in 2B and 2 C. ***p < 0.001, ****p < 0.0001. 3 C) Distribution of positive responses toward each ELISpot pool, as a proportion of overall positive responses. Groups combined to assess multiple prime (group 1 AAA_M, group 2 A_A_M, group 3 AAM), multiple boost (group 4 A_M_M_M), alternating vectors (group 5 AMA_M, group 6 AMAM, group 7 A_M_A_M) and single prime boost (group 5 + 6 AM, group 4 + 7 A_M).

Responses induced by ChAd63 ME-TRAP priming were of moderate magnitude and breadth, with positive responses to a median of 2 of the 7 ELISpot peptide pools corresponding to the ME-TRAP vaccine insert. Homologous ChAd63 ME-TRAP immunisations did not further broaden this response (Fig. 3A). MVA ME-TRAP vaccination broadened the response to a median of 5 positive pools, with a significant expansion in breadth over ChAd63 ME-TRAP at the second and third vaccination (Fig. 3B, Mann Whitney test p = 0.0002 and Fig. 3C p < 0.0001, respectively). To assess multiple-prime, multiple-boost and alternating vector regimens, the groups were combined and compared against single prime-boost responses. The distribution of positive responses was similar between regimens using multiple-prime, alternating vectors and single prime-boost, with a non-significant skew of responses to the C terminus of the vaccine insert with the multiple-MVA boost approach (Fig. 3D).

### Cellular immunogenicity by flow cytometry

Flow cytometry with intracellular cytokine staining (ICS) was performed 1 week and 4 weeks post MVA ME-TRAP vaccination. TRAP-specific CD4^+^ T cell responses were largely polyfunctional and consisted of 2 major phenotypes (IFN-γ^+^ IL-2^+^ TNFα^+^ or IL-2^+^ TNFα^+^). More variation was seen in the phenotype of TRAP-specific CD8^+^ T cells, with a greater proportion of monofunctional IFN-γ^+^ or TNFα^+^ cells detected compared with CD4^+^ T cells (data not shown).

**Figure 4:**
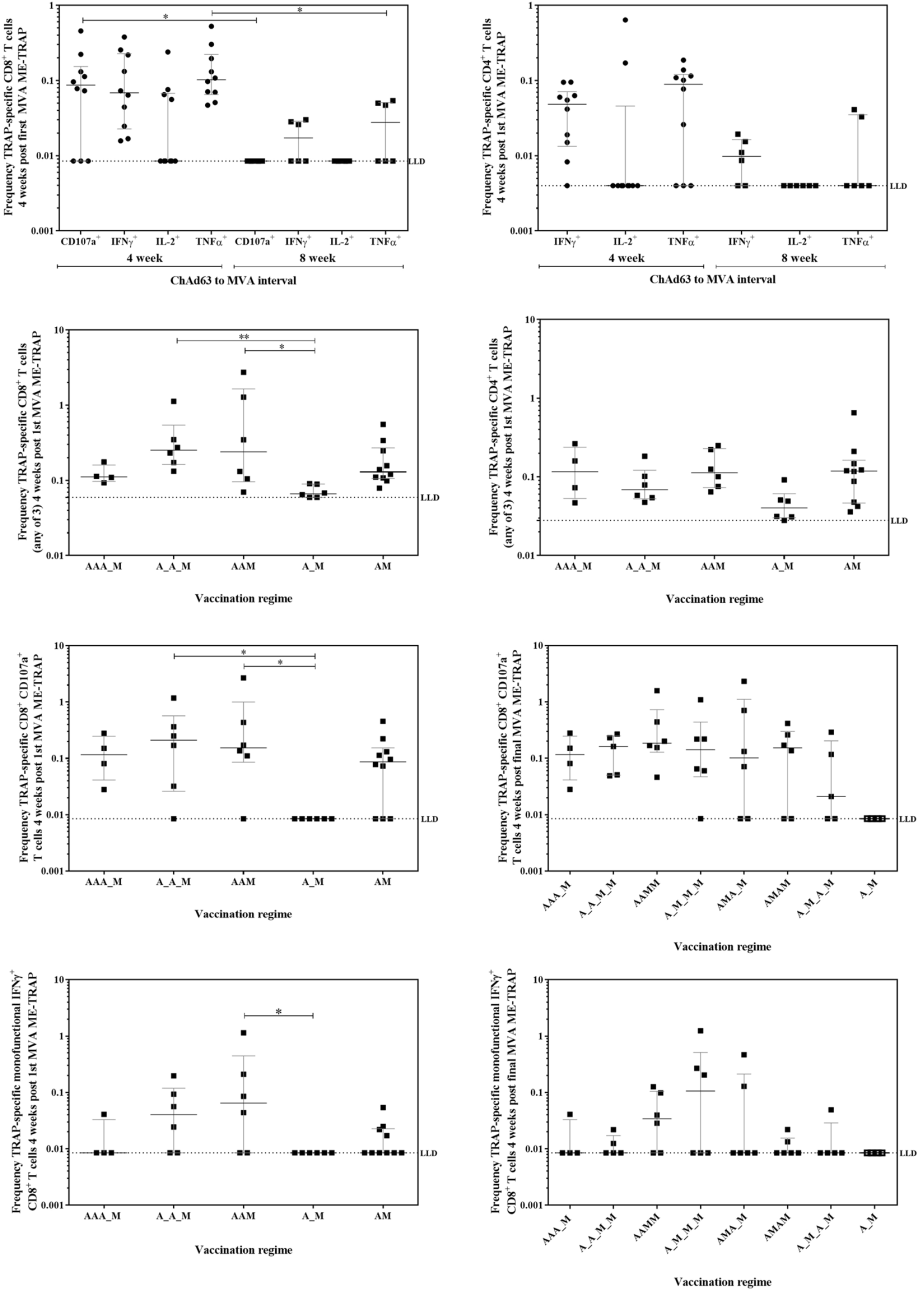
T cell responses measured by flow cytometry. **(A**) Frequency of TRAP-specific CD8^+^ T cells producing IFN-γ/IL-2/TNFα/expressing degranulation marker CD107a at 4 week post-MVA ME-TRAP for volunteers boosted at 4 weeks compared to 8 weeks post ChAd63 ME-TRAP. Kruskal-Wallis ANOVA with Dunn’s correction of paired cytokines at 4 vs 8 weeks, p < 0.0001. (**B**) Frequency of TRAP-specific CD4^+^ T cells producing IFN-γ/IL-2/TNFα at 1 week post-MVA ME-TRAP for volunteers boosted at 4 weeks compared to 8 weeks post ChAd63 ME-TRAP. No significant difference by Kruskal-Wallis ANOVA with Dunn’s correction of paired cytokines at 4 vs 8 weeks. (**C**) Frequency of CD8^+^ T cells producing producing any-of-3 cytokines from IFN-γ, IL-2 and TNFα 4 weeks post first MVA ME-TRAP vaccination. Kruskal-Wallis ANOVA between all groups with Dunn’s correction p = 0.0034. (**D**) Frequency of CD4^+^ cells producing any-of-3 cytokines from IFN-γ, IL-2 and TNFα 4 weeks post first MVA ME-TRAP vaccination. Kruskal-Wallis ANOVA between all groups with Dunn’s correction p = 0.0712. (**E**) Frequency of antigen-specific CD8^+^ CD107a^+^ T cells 4 weeks post first MVA ME-TRAP vaccination. Kruskal-Wallis ANOVA with Dunn’s correction against A_M group, p = 0.0198. (**F**) Frequency of antigen-specific CD8^+^ CD107a^+^ T cells 4 weeks post final MVA ME-TRAP vaccination. No significant difference by Kruskal-Wallis ANOVA between all 4-vaccination groups with Dunn’s correction p = 0.7699, and against A_M control group p = 0.0605. (**G**) Frequency of antigen-specific monofunctional CD8^+^ IFN-γ^+^ T cells 4 weeks post 1st MVA ME-TRAP vaccination. Kruskal-Wallis ANOVA with Dunn’s correction against A_M group, p = 0.0444. (**H**) Frequency of antigen-specific monofunctional CD8^+^ IFN-γ^+^ T cells 4 weeks post final MVA ME-TRAP vaccination. No significant difference by Kruskal-Wallis ANOVA between all 4-vaccination groups with Dunn’s correction p = 0.5846, and against A_M control group p = 0.3261. Medians with IQR displayed.

Expression of the degranulation marker CD107a (also known as LAMP-1) was used to identify CD8^+^ T cells with cytotoxic potential. Significantly higher frequencies of cytotoxic CD8^+^ T cells and CD8^+^ T cells producing TNFα were measured in volunteers immunised with a 4- week ChAd63 ME-TRAP to MVA ME-TRAP interval, compared with an 8-week interval (Fig. 4A Kruskal-Wallis ANOVA with Dunn’s correction on paired samples grouped by interval length, p < 0.001). A similar but non-significant trend was also measured in TNFα-secreting CD4^+^ T cells (Fig. 4B). Any-of-3 cytokine analysis (IFN-γ, IL-2 or TNFα) of the 5 different priming regimens showed a significantly higher magnitude of antigen-specific CD8^+^ T cells in the 2 regimens with a double ChAd63 prime (group 2 A_A_M and group 3 AAM) compared to the standard 8 week prime-boost regimen (group 4 and 7 combined, A_M), (Fig. 4C Kruskal-Wallis ANOVA with Dunn’s correction for multiple comparisons between all groups, p = 0.003). Again, a similar but non-significant trend was seen with TRAP-specific CD4^+^ T cells (Fig. 4D). No differences were measured in CD4^+^ or CD8^+^ T cell responses 1 week post MVA ME-TRAP vaccination (data not shown).

The correlates of protection previously measured with the A_M regimen, CD107a expression and IFN-γ production by CD8^+^ T cells (IFN-γ^+^, IL-2^−^, TNFα^−^) were compared after A_M to each of the vaccination groups. One week post first MVA ME-TRAP vaccination, frequencies of monofunctional IFN-γ^+^ CD8^+^ T cells were comparable between all priming regimens, as was CD107a expression (data not shown). By 4 weeks post first MVA ME-TRAP vaccination, the 2 regimens with a double ChAd63 prime (group 2 A_A_M and group 3 AAM) had significantly higher TRAP-specific CD8^+^ CD107a^+^ T cells (Fig. 4E, Kruskal-Wallis ANOVA with Dunn’s correction against A_M group, p = 0.0198). Significantly higher monofunctional IFNγ^+^ CD8^+^ T cells were also measured in group 3 AAM (Fig. 4G, Kruskal-Wallis ANOVA with Dunn’s correction compared with A_M group, p = 0.04). These differences were not measured following the final MVA ME-TRAP vaccination (Fig. 4F,H).

### Antibody immunogenicity by ELISA

**Figure 5:**
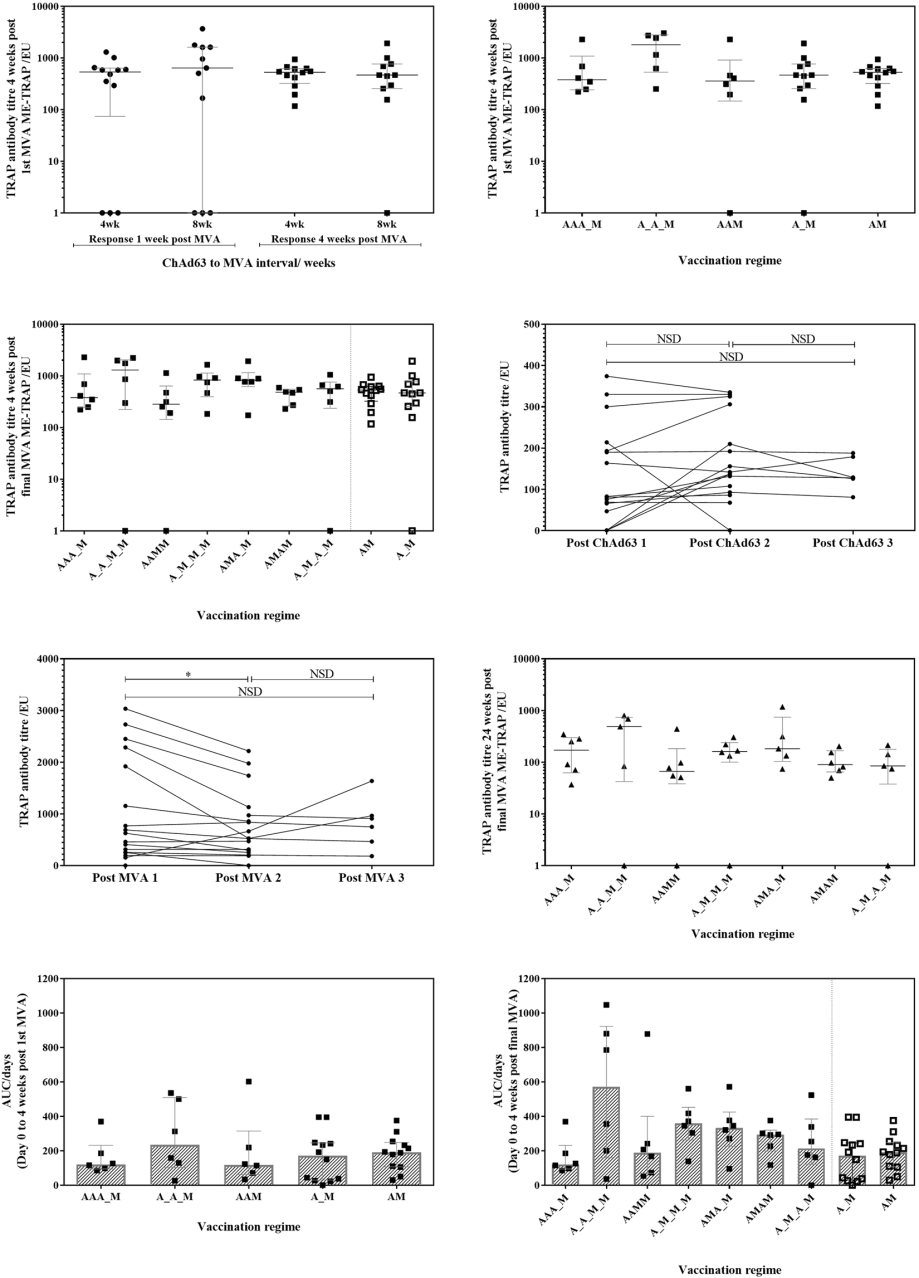
TRAP ELISA responses. (**A**) Responses 1 week and 4 weeks post-MVA ME-TRAP responses for volunteers boosted at 4 weeks compared to 8 weeks post ChAd63 ME-TRAP. No significant difference at 1 or 4 weeks post boost, Mann Whitney test p = 0.3847 and 0.9754, respectively. (**B**) Responses 4 weeks post-MVA ME-TRAP response following single, double and triple ChAd63 ME-TRAP priming vaccination(s), with single MVA ME-TRAP boost. No significant difference between groups by Kruskal-Wallis ANOVA between all groups with Dunn’s correction p = 0.1597. (**C**) Response 4 weeks post final MVA ME-TRAP. No significant difference between groups by Kruskal-Wallis ANOVA between all groups with Dunn’s correction p = 0.4277. (**D**) Peak ELISA response following each ChAd63 ME-TRAP response per volunteer. Trend to higher responses after 2nd ChAd63 ME-TRAP vaccination compared to 1st ChAd63 ME-TRAP vaccination p = 0.0735 by Wilcoxon matched pairs test. Trend to higher responses after 1st ChAd63 ME-TRAP compared to 3rd ChAd63 ME-TRAP p = 0.0585. No significant difference between ChAd63 ME-TRAP 2 and ChAd63 ME-TRAP 3 p = 0.2932. (**E**) Peak ELISA response following each MVA ME-TRAP vaccination. Significantly higher response after 2nd MVA ME-TRAP compared to 1st MVA ME-TRAP by Wilcoxon matched pairs test between MVA ME-TRAP 1 and MVA ME-TRAP 2, p = 0.0103. No significant differences between MVA ME-TRAP 2 and MVA ME-TRAP 3 p = 1.000 or MVA ME-TRAP 1 and MVA ME-TRAP 3 p = 0.6250. (**F**) TRAP antibody titre 24 weeks post-last vaccination. No significant difference between groups by Kruskal-Wallis ANOVA with Dunn’s correction p = 0.466. (**G**) Individual AUC response for day 0 to 4 weeks post 1st MVA ME-TRAP vaccination. No significant difference between groups by Kruskal-Wallis ANOVA with Dunn’s correction p = 0.7932, (**H**) Individual AUC response for day 0 to 4 weeks post final MVA ME-TRAP vaccination. No significant difference between groups by Kruskal-Wallis ANOVA with Dunn’s correction between all groups, p = 0.0712. Median with IQR displayed.

TRAP-specific IgG antibody titres 1 and 4 weeks after boosting with MVA were not significantly different between a 4- or 8- week ChAd63 ME-TRAP to MVA ME-TRAP interval (Fig. 5A, Mann Whitney test p = 0.3847 and 0.9754, for 1 and 4 weeks respectively). ChAd63 ME-TRAP vaccination induced modest TRAP-specific total IgG antibody responses (median 78 ELISA units [EU] across all groups at day 28). Additional ChAd63 ME-TRAP vaccinations at either 4 or 8 week intervals before a later MVA ME-TRAP boost did not significantly increase the peak antibody titre compared with groups receiving only one ChAd63 vaccination (Fig. 5B, Kruskal-Wallis ANOVA between all groups with Dunn’s correction p = 0.16). Equally, there was no difference in anti-TRAP antibody titre following the 4-vaccination schedules with all group titres comparable to the previously published 8 week prime-boost regimen (Fig. 5C, Kruskal-Wallis ANOVA between all groups with Dunn’s correction p = 0.43).

The kinetics of homologous administration of ChAd63 in group 1 (AAA_M), group 2 (A_A_M_M) and group 3 (AAMM) saw a trend towards a higher TRAP antibody titre following the second and third ChAd63 ME-TRAP vaccination compared to the first (Fig. 5D, Wilcoxon matched pairs signed rank test ChAd63 ME-TRAP 1 and ChAd63 ME-TRAP 2 p = 0.0735, ChAd63 ME-TRAP 1 and ChAd63 ME-TRAP 3 p = 0.0585, ChAd63 ME-TRAP 2 and ChAd63 ME-TRAP 3 p = 0.2932). A 7-fold increase in antibody titre from peak post-prime to peak post-boost across all individuals was measured (median peak titre 522 EU, data not shown). Peak antibody responses in all but group 5 occurred after the first MVA ME-TRAP, and subsequent MVA vaccinations did not re-boost titres to an average higher than the original peak. Paired analysis of peak responses after consecutive MVA ME-TRAP vaccination in group 2 (A_A_M_M), group 3 (AAMM) and group 4 (A_M_M_M) showed no significant boosting in TRAP antibody response with subsequent MVA ME-TRAP vaccinations, with a significantly higher TRAP antibody response following the first MVA ME-TRAP vaccination compared to the second (Fig. 5E, Wilcoxon matched-pairs signed rank test between MVA ME-TRAP 1 and MVA ME-TRAP 2 p = 0.01, MVA ME-TRAP 1 and MVA ME-TRAP 3 p = 0.63 or MVA ME-TRAP 2 and MVA ME-TRAP 3 p = 1.0). Antibody responses waned over time and were comparable to pre-boost titres by 24 weeks post-last vaccination (WPLV). Titres at this time point were not significantly different between groups (Fig. 5F, Kruskal-Wallis ANOVA with Dunn’s correction p = 0.47).

AUC analysis of antibody responses revealed no significant difference between a single, double or triple ChAd63 prime followed by a single MVA boost (Fig. 5G, Kruskal-Wallis ANOVA with Dunn’s correction p = 0.79), or between any of the 4-vaccination or 2-vaccination regimens (Fig. 5H, Kruskal-Wallis ANOVA with Dunn’s correction p = 0.07). Median time courses of TRAP-specific IgG responses are shown in Fig. 2.

### Anti-vector immunity

**Figure 6:**
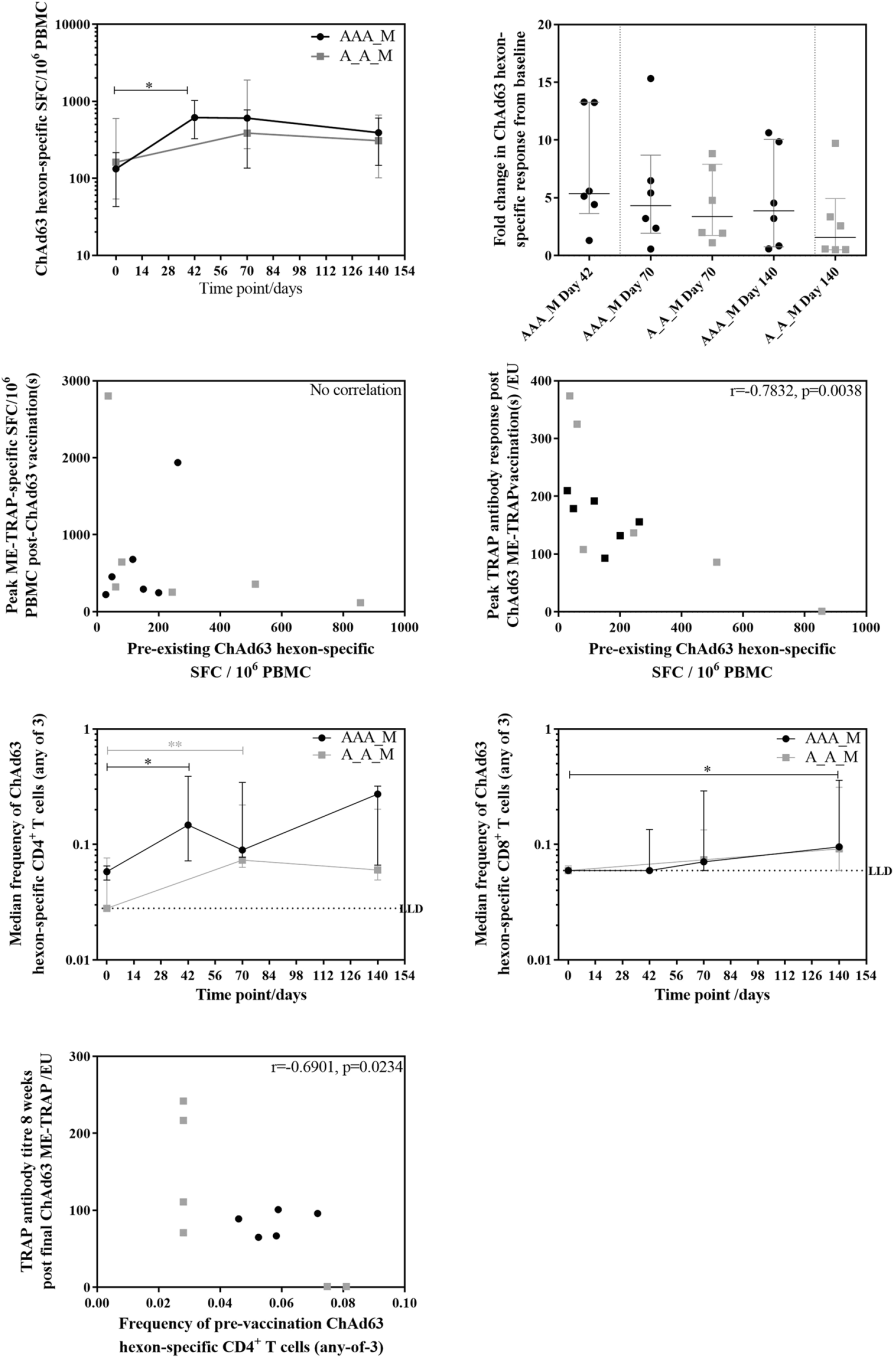
Vector-specific immunity against ChAd63. (**A**) ChAd63 hexon-specific response time course by *ex vivo* IFN-γ ELISpot following triple ChAd63 ME-TRAP priming and double ChAd63 ME-TRAP priming. Friedman test with Dunn’s correction for time course within group p = 0.0130, Kruskal-Wallis test with Dunn’s correction for between group analysis p = 0.0760. (**B**) Fold change in anti-ChAd63 hexon response by *ex vivo* IFN-γ ELISpot. No significant difference between groups or time points by Kruskal-Wallis ANOVA with Dunn’s correction, p = 0.3463. (**C**) Correlation between pre-vaccination ChAd63 hexon *ex vivo* IFN-γ ELISpot responses and peak post-ChAd63 *ex vivo* IFN-γ ELISpot responses to ME-TRAP by Spearman r. r = −0.2378, p = 0.4573. (**D**) Correlation between pre-vaccination ChAd63 hexon *ex vivo* IFN-γ ELISpot responses and peak post-ChAd63 TRAP antibody titre by Spearman r. r = −0.7832, p = 0.0038. (**E**) Frequency time course of ChAd63-specific (any-of-3) CD4^+^ T cells, Friedman test with Dunn’s correction against pre-vaccination within group 1 p = 0.0167, group 2 p = 0.0001, no significant difference between groups Kruskal-Wallis test with Dunn’s correction between time points p = 0.0160). (**F**) Frequency time course of ChAd63-specific (any-of-3) CD8^+^ T cells, Friedman test with Dunn’s correction against pre-vaccination within group 1 p = 0.0065, group 2 p = 0.3086, no significant difference between groups Kruskal-Wallis test with Dunn’s correction between time points p = 0.0391). (**G**) Correlation between pre-vaccination ChAd63 hexon-specific CD4^+^ T cells and post-ChAd63 TRAP antibody titre at day 112 by Spearman r. r = −0.6901, p = 0.0234. Black = group 1, grey = group 2.

Pre-vaccination T cell responses to the ChAd63 hexon were positive in 8 /12 (67%) of volunteers by *ex vivo* IFN-γ ELISpot, were generally low in magnitude (median 133 SFC) and were comparable between groups 1 and 2. Post-vaccination responses were also comparable between groups. Individual ELISpot responses to the ChAd63 hexon increased significantly in group 1 after 2 doses of ChAd63 administered with a 4-week interval (Fig. 6A, Friedman test with Dunn’s correction for time course within group p = 0.013, Kruskal-Wallis test with Dunn’s correction for between group analysis p = 0.08). The overall anti-vector fold increase from day 0 was no greater in the triple ChAd63 ME-TRAP group compared to the group receiving two priming doses in the same 8-week period (Fig. 6B Kruskal-Wallis AVOVA with Dunn’s correction, p = 0.35).

There was no correlation between pre-vaccination T cell response to the ChAd63 hexon and the peak post-ChAd63 ME-TRAP T cell response to the vaccine insert (Fig. 6C, group 1 and 2 combined). A negative correlation was measured between pre-existing T cell responses to the ChAd63 hexon and peak TRAP antibody titre post-ChAd63 ME-TRAP vaccination(s) (Fig. 6D, Spearman r = −0.7832, p = 0.0038). Boosting with MVA ME-TRAP overcame this effect, with no correlation measured between pre-existing T cell responses to the ChAd63 hexon and TRAP-specific antibody titres post MVA ME-TRAP. The negative correlation was not enhanced with vaccine-induced ChAd63 hexon-specific T cells, with no correlation between the hexon T cell response following multiple ChAd63 ME-TRAP vaccinations at day 70 and TRAP antibody titre at day 84 (data not shown).

Intracellular cytokine staining revealed that both CD4^+^ and CD8^+^ cytokine-secreting T cells contribute to the ChAd63-hexon specific T cell response, with significant boosting of ChAd63 hexon-specific CD4^+^ T cells in groups 1 and 2 after 2 doses of ChAd63 ME-TRAP (Fig. 6E, Friedman test with Dunn’s correction within group 1 p = 0.0167, group 2 p = 0.0001) and ChAd63 hexon-specific CD8^+^ T cells in group 1 only (Fig. 6F, Friedman test with Dunn’s correction within group 1 p = 0.007, group 2 p = 0.31). Anti-vector CD4^+^ and CD8^+^ T cell responses between groups were comparable at each time point (data not shown). No correlations were measured between anti vector CD4^+^ or CD8^+^ T cell response and the T cell response to the vaccine insert. A negative correlation was measured between pre-vaccination CD4^+^ ChAd63-specific T cells and TRAP antibody titres 8 weeks post final ChAd63 ME-TRAP vaccination (Fig. 6G Spearman r = −0.69, p = 0.02).

**Figure 7:**
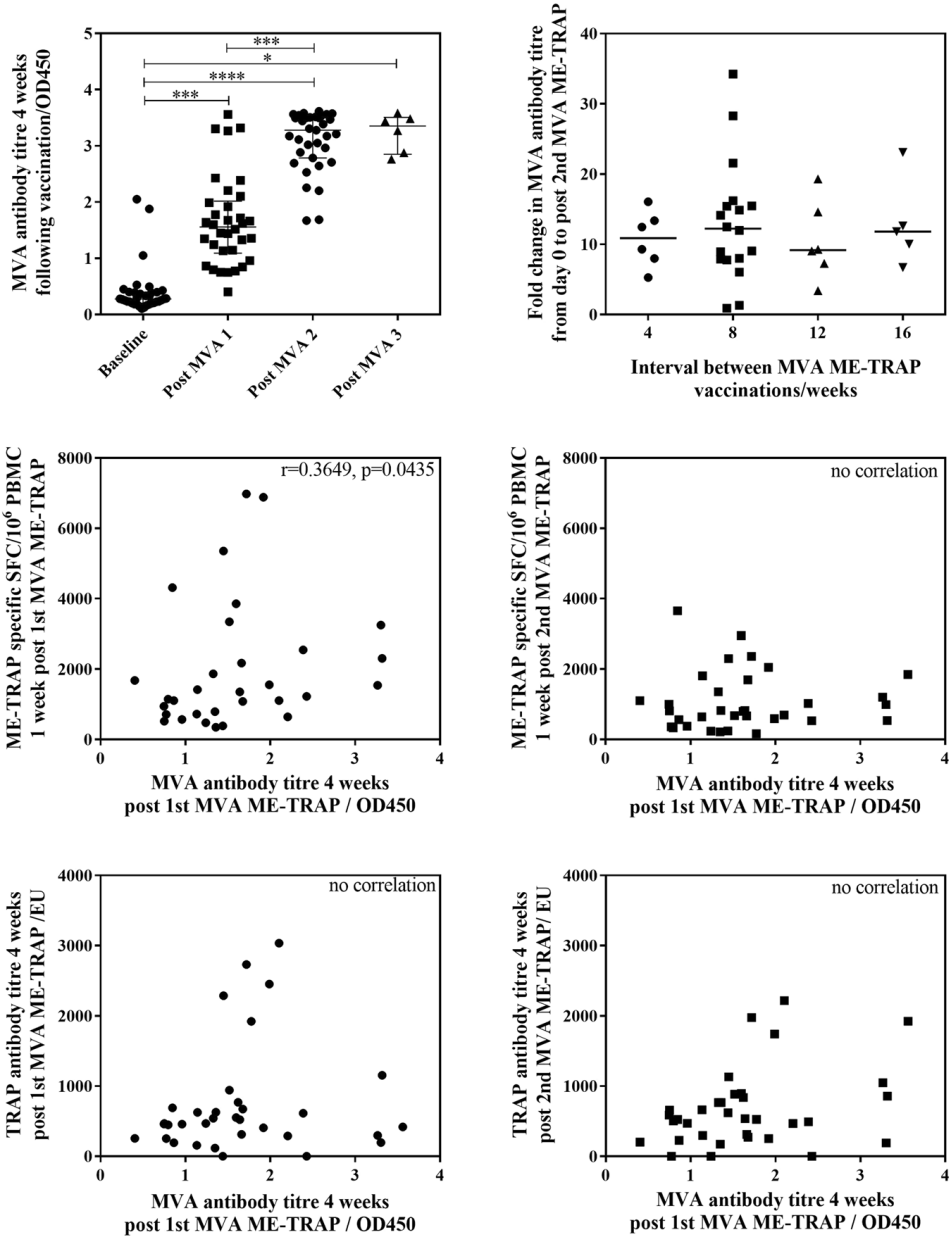
Vector-specific immunity against MVA. (**A**) Anti-MVA ELISA OD 28 days post MVA ME-TRAP for groups 2–7. Friedman test with Dunn’s comparison between multiple time points. P < 0.0001. (**B**) Fold change in anti-MVA ELISA OD stratified by interval between MVA ME-TRAP vaccinations. No significant difference in fold change with different interval length by Kruskal-Wallis ANOVA between all groups with Dunn’s correction p = 0.9099. (**C**) Correlation anti-MVA antibody titre 4 weeks post 1st MVA ME-TRAP and T cell response to ME-TRAP 1 week post- 1st MVA ME-TRAP (Spearman r = 0.3649, p = 0.0435). (**D**) Correlation anti-MVA antibody titre 4 weeks post 1st MVA ME-TRAP and T cell response to ME-TRAP 1 week post- 2nd MVA ME-TRAP (Spearman r = 0.1260, p = 0.4847. (**E**) Correlation anti-MVA antibody titre 4 weeks post 1st MVA ME-TRAP and TRAP antibody titre 4 weeks post 1st MVA ME-TRAP vaccination (Spearman r = 0.1754, p = 0.3210). (**F**) Correlation anti-MVA antibody titre 4 weeks post 1st MVA ME-TRAP and TRAP antibody titre 4 weeks post 2nd MVA ME-TRAP vaccination (Spearman r = 0.2796, p = 0.1093).

Anti-MVA antibody levels were measured using an ELISA to the MVA WR113/D8L protein^26^ following each MVA ME-TRAP vaccination in groups 2–7. Analysis of combined groups saw a significant increase in anti-MVA antibodies after the first MVA ME-TRAP vaccination, and again after the second vaccination (Fig. 7A, Friedman test with Dunn’s comparison between multiple time points p < 0.0001). The fold change from day 0 to post-second MVA ME-TRAP was not affected by the interval between MVA ME-TRAP vaccinations (Fig. 7B). A positive correlation was measured between anti-MVA antibodies 4 weeks post first MVA ME-TRAP vaccination and ME-TRAP-specific T cells 1 week post first MVA ME-TRAP vaccination (Fig. 7C, Spearman r = 0.36, p = 0.04), with no correlation following the second MVA ME-TRAP vaccination (Fig. 7D). No correlation was measured between MVA antibodies 4 weeks post first MVA ME-TRAP vaccination and TRAP antibody titre following the first and second MVA ME-TRAP vaccination (Fig. 7E,F, respectively). No correlations were measured between anti-MVA antibodies and TRAP-specific T cells or antibodies following the second or third MVA ME-TRAP vaccination (data not shown).

## Discussion

The safety profiles of ChAd63 ME-TRAP and MVA ME-TRAP observed in this trial were similar to previous clinical studies of these vaccines, with MVA ME-TRAP more reactogenic than ChAd63 ME-TRAP^27–30^. There was no difference in vaccine-related adverse event profiles with repeated homologous administration of ChAd63 ME-TRAP (group 1, 2 and 3) or MVA ME-TRAP (group 2, 3 and 4) or with the alternating administration of each vector (group 5, 6 and 7), as compared with previous studies. Therefore, the number of vaccinations and the timing, frequency and order of vaccine administration did not appear to play a significant role in determining the adverse event profile of the novel schedules tested.

Although the group sizes in this Phase I clinical vaccine trial were small, we have found no evidence to suggest that reducing the prime-boost interval from 8 to 4 weeks has a negative impact on induction of TRAP-specific antibodies or T cells. This is consistent with published data using ChAd63 and MVA viral vectors at shorter intervals for vaccination against Ebola virus disease in a subsequently conducted trial^31^. In that Ebola virus vaccine trial, higher frequencies of antigen-specific T cells were measured with a shortened prime-boost interval, with the interval reduced to as little as one week.

CD107a and b are markers of CD8^+^ T cell degranulation that have been shown to mediate cytolysis activity in an antigen-specific manner^32^. Following an 8 week ChAd63 ME-TRAP and MVA ME-TRAP prime-boost regimen, TRAP-specific CD8^+^ CD107a^+^ T cells measured 24 weeks post boost and monofunctional TRAP-specific CD8^+^ T cells producing IFN-γ measured 14–21 days post boost correlated with a higher degree of protection against CHMI^20^. In this study, both of these parameters were similar across all 2-vaccination and 4-vaccination groups at 1 week post first MVA and at 4 weeks post final MVA. However, at a previously unreported time point of 4 weeks post-first MVA ME-TRAP vaccination, significantly higher frequencies of TRAP-specific degranulating CD8^+^ T cells were measured in the 2 groups with a double ChAd63 prime (group 2 and 3) compared with the standard 8 week prime-boost regimen. This improvement over the 8-week prime-boost regimen was also measured in monofunctional CD8^+^ IFN-γ^+^ T cells in group 3. Together these findings suggest that greater vaccine efficacy might be achieved by including an additional ChAd63 ME-TRAP vaccination, with a 4- or 8-week vaccination interval (AAM or A_A_M) than with the 8-week ChAd63 MVA ME-TRAP prime-boost schedule (A_M). With significantly higher frequencies of CD8^+^ TNFα^+^ and CD8^+^ CD107a^+^ T cells following the AM regimen compared with the A_M regimen, schedules with four-week intervals (AM or AAM) would fit well with the standard 6, 10, 14-week Expanded Programme on Immunization (EPI) schedule that is used for infant vaccinations in most countries where malaria is highly endemic.

CD8^+^ T cell responses following vaccination are of particular importance, due to their proven association with protection at the liver stage of infection in mice^33–35^ and humans^20^. Immunogenicity of the 8-week regimen is improved if a double ChAd63 prime is used, thus giving flexibility to the regimen, without compromising immunogenicity.

Consistent with previous macaque data using the same viral vectors, MVA ME-TRAP vaccination further broadens the response induced by ChAd63 ME-TRAP vaccination, and multiple MVA ME-TRAP boosts do not significantly enhance humoral or cellular immunogenicity beyond the magnitude of the first MVA ME-TRAP vaccination^25^. However additional dose(s) of MVA boosted individual peak T cell immunogenicity in over 1/3 of volunteers, which may be an important consideration when tested in larger numbers of volunteers, or in populations where co-infection with malaria or other factors can reduce cellular responses to vaccination^36–39^.

The breadth of T cell response to the vaccine insert is comparable with previous non-human primate studies with respect to ChAd63 ME-TRAP and MVA ME-TRAP vaccinations and multiple MVA ME-TRAP boosting vaccinations^25^. The reported response breadth of 2 pools measured after ChAd63 ME-TRAP vaccination and 5 pools after MVA ME-TRAP vaccination is comparable to or broader than previously reported in vaccinated populations in the UK and Africa^10,23^. With no single immunodominant epitope in ME-TRAP, T-cell responses directed towards a wider variety of epitopes may confer enhanced protection^40^.

A previous clinical vaccine trial assessing vaccine efficacy of ChAd63.MVA ME-TRAP against malaria infection in Kenyan adults demonstrated that a large proportion of the ELISpot response is directed towards TRAP T9/96 pools 2 (TT11-20) and 3 (TT21-30). T cell responses to TRAP pool 3 were significantly associated with protection against natural malaria infection^21^ and all regimens tested in this study directed 10–20% of positive responses to this region of the TRAP antigen.

Anti-TRAP antibody titres in this trial were generally lower than previously measured with the ChAd63.MVA ME-TRAP regimen^41^. However, across this trial, responses were comparable for individuals boosted at either 4 or 8 weeks, and between 2- and 4- vaccination regimens. Despite a negative correlation between TRAP antibodies and pre-existing ChAd63-specific T cells, regimens with a single, double or triple dose of ChAd63 ME-TRAP yielded comparable TRAP antibody titres. Unlike T cell immunogenicity, multiple MVA boosts did not re-boost TRAP antibody titres to the same level as the first, with responses to the second MVA ME-TRAP significantly lower than after the first.

ICS data suggested that this negative correlation between pre-existing ChAd63-hexon specific T cells measured by ELISpot and post prime TRAP antibody titre was driven by a ChAd63 hexon-specific CD4^+^ T cell response. Because prior exposure to ChAd63 itself is extremely unlikely in UK volunteers, we assume that the pre-existing T cells represent cross-reactivity with and prior exposure to another human adenovirus. While the first dose of ChAd63 increases the frequency of ChAd63 hexon-specific T cells, these vector-specific T cells are apparently not detrimental to vaccine-induced T cell or antibody immunogenicity towards the vaccine insert. Furthermore, post-vaccination ChAd63 hexon-specific T cells frequencies were comparable between the 2 groups at each time point, suggesting that additional doses of ChAd63 ME-TRAP after the initial priming dose do not increase the magnitude of the T cell response against the vector, however a 3rd dose may prolong the persistence of these cells. One possible explanation for this is that pre-existing, naturally-acquired, anti-vector T cell immunity is of a different quality to that of vaccination-acquired, anti-vector T cell immunity, as demonstrated by negative correlations between the TRAP antibody response and day 0 anti-vector T cell responses, followed by loss of this correlation once ChAd63 is administered as a vaccine. Firstly, pre-existing ChAd63 hexon-specific T cell responses are likely due to cross reactivity with human adenovirus exposure, and not pre-existing ChAd63 exposure. Secondly, wild-type adenovirus and genetically modified vaccine vectors differ in their replication competency. The implications in this setting are that multiple homologous administrations of ChAd63 do not impair immunogenicity to ME-TRAP following vaccination, but that pre-existing T cells to the adenoviral vector may impact upon vaccine immunogenicity, particularly on the antibody response to the vaccine insert.

The positive correlation between anti-MVA antibodies and TRAP-specific T cell response following the first MVA vaccination is analogous to the trend previously measured towards higher peak T cell responses to the TRAP insert in those volunteers with anti-vector antibodies^20^. TRAP-specific T cells were re-boosted by subsequent homologous MVA ME-TRAP vaccinations to levels comparable to that of the first boost, which is similar to observations in a previous macaque study^25^. It was unknown whether multiple administrations would enhance regimen immunogenicity or impair it through induced anti-vector immunity: with no negative correlations measured between anti-MVA antibodies and TRAP-specific T cell or antibody responses following double and triple administration of MVA ME-TRAP, we conclude that multiple MVA vaccinations do not induce detrimental anti-vector immunity.

In summary, a novel vaccination regimen with an additional ChAd63 ME-TRAP priming vaccination may achieve greater protection than the 8 week ChAd63.MVA ME-TRAP prime-boost schedule. This regimen does not induce detrimental anti-vector T cell immunity. The flexibility demonstrated in this vaccination regimen would facilitate incorporation into the WHO Expanded Programme on Immunization, whereby childhood immunisations are administered at intervals ranging from 4 to 12 weeks. Such flexibility also permits combining this approach with other partially protective malaria vaccine regimens using protein-based subunit vaccines plus adjuvant.

## Materials and Methods

### Data Availability

The datasets generated and analysed during the current study are available from the corresponding author on reasonable request.

### Participants

The study was conducted at the Centre for Clinical Vaccinology and Tropical Medicine, University of Oxford, Oxford, UK and the Wellcome Trust Clinical Research Facility, Southampton, UK. Healthy, malaria-naïve males and non-pregnant females aged 18–50 were invited to participate and were enrolled upon written, informed consent, according to the principles of the 2008 Declaration of Helsinki and in accordance with Good Clinical Practice.

### Study Design

Novel prime-boost schedules were assessed in 7 groups of 6 volunteers. Each volunteer received four vaccinations intramuscularly into the deltoid region of the arm. Schedules are summarised in Table 1, consisting of intramuscular administration of ChAd63 ME-TRAP 5 × 10^10^ v.p. and MVA ME-TRAP 2 × 10^8^ p.f.u. Blood sampling was performed at several follow up time points, to assess both safety and immunogenicity. A Local Safety Monitor provided safety oversight and GCP compliance was externally monitored.

### Ethical Approval

The study protocol was approved by the Oxford Research Ethics Committee (reference 10/H0604/96) and the Medicines and Healthcare Products Regulatory Agency (EudraCT number: 2010-023824-26). The trial was registered with ClinicalTrials.gov on 31^st^ May 2011 (Ref: NCT01364883).

### ChAd63 ME-TRAP and MVA ME-TRAP vaccines

Manufacture of ChAd63 ME-TRAP by the Clinical Biomanufacturing Facility, University of Oxford and MVA ME-TRAP by IDT Biologika, Rosslau, Germany were performed under Good Manufacturing Practice conditions as previously described [19].

### PBMC Preparation

Blood samples for PBMC separation were collected in lithium heparin Vacutainer® blood collection systems (Becton Dickinson, UK) and separated on a centrifugation gradient using Lymphoprep® (Axis Shield) within 6 hours of venepuncture. All protocols and record keeping were performed in compliance with Good Clinical Practice.

### Peptides for T cell assays

Peptides were manufactured by NEOBiolab (Cambridge, MA, USA) to >70% purity. Peptides covering the full length of Thrombospondin-Related Adhesion Protein were 20 amino acids in length and overlapped by 10 amino acids, as previously described^20,21^). Peptides to the multi-epitope string comprising CD4^+^ and CD8^+^ T cell epitopes from *P. falciparum* (described in detail in ref.^19^) were between 8 and 20 amino acids in length depending on the epitope and identical to those used previously^20,21^. Hexon peptides were 20 amino acids in length and were pooled according to peptides specific to ChAd63 and common to both ChAd63 and AdHu4 adenovirus. Peptides were reconstituted in DMSO at 50–200 mg/mL and pooled for ELISpot and flow cytometry analyses.

### *Ex vivo* interferon-γ ELISpot

Assays were performed using Multiscreen IP ELISpot plates (Millipore) coated using 10 μg/mL human IFN-γ and developed using SA-ALP antibody kits (both Mabtech) and BCIP NBT-plus chromogenic substrate (Moss Inc.). Cells were cultured in RPMI (Sigma) containing 1000 units/mL penicillin, 1 mg/mL streptomycin and 10% heat-inactivated, sterile-filtered foetal calf serum, previously screened for low reactivity (Labtech International) for 18–20 hours. Antigens were tested in duplicate, with 2.5 × 10^5^ peripheral blood mononuclear cells (PBMC) added to each well of the *ex vivo* ELISpot plate in a final volume of 100 μL, assayed in 6 pools of 7–10 peptides at 10 μg/mL for TRAP. Responses to the multiple epitope string were assayed in duplicate using a single peptide pool at 10 μg/mL. Responses were averaged across duplicates, responses in unstimulated (negative control) wells were subtracted and responses in individual pools summed for the TRAP antigen, plus ME. Staphylococcal enterotoxin B (0.02 μg/ml) and phytohaemmagglutinin-L (10 μg/ml) were pooled and used as a positive control. Plates were counted using an AID automated ELISpot counter (AID Diagnostika GmbH, algorithm C), using identical settings for all plates and counts were adjusted only to remove artefacts. ELISpot QC passed assays with >800 spot-forming cells per million PBMC (SFC) in at least one of the positive control wells, and <80 SFC or <50% of the antigen-specific response in the negative control wells. Responses of >83 SFC after subtraction of background were considered positive (median of 10 SFC plus 2 standard deviations of the negative controls across all assays). The lower limit of detection for the assay was 28 SFC/10^6^ PBMC for ME-TRAP ELISpots and 16 SFC/10^6^ PBMC for hexon ELISpots. Hexon ELISpots were performed on total pool hexon peptides (summed ChAd63-specific and common peptides).

### Flow Cytometry with ICS

1.25 × 10^6^ freshly isolated PBMC in 1 ml of medium containing anti-CD28 and anti-CD49d (1 μg/ml, eBioscience) and CD107a-PeCy5 (1:100, eBioscience) were stimulated in 5 ml polystyrene FACS tubes for 18–20 hours at 37 °C in 5% CO2. For TRAP-specific responses, PBMC were stimulated with a single pool of 56 peptides spanning the T9/96 strain of the TRAP antigen (2 μg/ml). For anti-ChAd63 hexon responses, peptides spanning the ChAd63 hexon and with no homology to AdHu4 (ChAd63-specific) were tested (2 μg/ml). Unstimulated PBMC and Staphylococcal enterotoxin B (SEB, Sigma, 1 μg/ml) stimulated PBMC were run in parallel as negative and positive controls, respectively. Brefeldin A (30 μg/mL, eBioscience) and monensin (1:1000, eBioscience) were added for the last 16–18 hours of stimulation. After stimulation, cells were washed in PBS containing 0.1% bovine serum albumin (Sigma) and 0.01% sodium azide (Sigma), and PBMC separated from an unstimulated tube to act as an unstained control. PBMC were then incubated with Aqua LIVE/DEAD cell discrimination dye (1:200, Thermofisher) at 4 °C for 10 minutes and surface-stained at 4 °C for a further 30 minutes with anti-human CD4 Qdot® 605 (1:50, Life Technologies) and CD14- and CD19-Pacific Blue (both 1:50, eBioscience). After incubation in Fix/Perm buffer for 30 minutes (BD Biosciences), intracellular staining was performed at 4 °C for 30 minutes with CD3-Alexa Fluor 700 (1:50, eBioscience), CD8- APC-Alexa Fluor 780 (1:10), IFN-γ-FITC (1:25), IL-2-PE (1:50) and TNFα-PE-Cy7 (1:50, all eBioscience). Samples were washed in Perm Wash (1:10, BD Biosciences) and fixed in 1% paraformaldehyde. Samples were immediately acquired on an LSRII cytometer (BD Biosciences) using FACSDiva v6.2 (BD Biosciences), with the relevant single fluorochrome compensation controls and photo multiplier tube voltages set by daily acquisition of Cytometer Setup and Tracking beads (BD Biosciences). A minimum of 10,000 CD4^+^ and CD8^+^ cells were collected per sample. Data were analysed using FlowJo v10.0.7 (Treestar Inc). Cells were gated on lymphocytes, singlets, live cells, CD3^+^CD14^−^CD19^−^, CD4^+^CD8^−^ or CD8^+^CD4^−^, then assessed for IFN-γ, IL-2, TNFα secretion, combinations of these cytokines and for CD107a expression. A sample gating strategy is provided in Figure S1.

Responses to peptide were determined after subtraction of the response in the unstimulated control for each sample. A cytokine response is deemed positive if greater than the background response, is representative of >50 cells and is above the lower limit of detection for CD4^+^ and CD8^+^ T cells (calculated by [1/lowest number of CD4^+^ or CD8^+^ cells acquired in antigen-stimulated or unstimulated samples] × 100). A sample passed positive QC if cytokine production for at least 1 cytokine was >1% in SEB stimulated cells.

### Anti-TRAP ELISA

Serum was taken for antibody analysis at D0, D28, D56, D84, D112 and 24 WPLV for all volunteers. Serum was additionally taken at 1 and/or 4 weeks after MVA vaccinations, according to the vaccination schedule in each group. Antibody responses to TRAP were measured using total IgG Enzyme Linked Immunosorbent Assay (ELISA) as previously published^41^. Briefly, Nunc-Immuno 96-well plates were coated with 0.5 µg/mL of TRAP antigen in carbonate-bicarbonate coating buffer at 4 °C overnight. Plates were washed 6 times with PBS-Tween (PBS/T) and blocked with 1% BSA in PBS with 0.05% Tween 20 for 1 hour at room temperature (RT). Serum dilutions were made using 0.2% BSA in PBS/T, added to the plate in triplicate and incubated for 2 hours at RT. Secondary antibody (goat anti-human whole IgG conjugated to alkaline phosphatase, Sigma) was added at a dilution of 1:1000 in PBS/T with 0.2% BSA for 1 hour at RT. Plates were washed a final time and developed using p-NitrophenylPhosphate (Sigma) in diethanolamine buffer (Pierce). Optical density (OD) was read at 405 nm on an ELx800 microplate reader (Biotek) with Gen5 software (version 1.10).

A positive reference pool of TRAP-positive serum was used to form a standard curve on each plate. Reference serum was added in duplicate at an initial dilution of 1:100 (in PBS/T 0.2% BSA) and diluted 2-fold 10 times. The initial dilution was assigned an arbitrary 20 antibody units and OD values were fitted to a 4-parameter logistic curve. A 1:800 dilution (in PBS/T 0.2% BSA) of this reference pool was also included in triplicate on each plate as an internal control. ELISA units were calculated from their OD values using the parameters estimated from the standard curve.

### Anti-MVA ELISA

Anti-MVA ELISAs were conducted using serum taken at baseline (Day 0) and 4 weeks after each MVA vaccination for all volunteers. Pre-coated and pre-blocked 96-well ELISA plates were kindly donated by Dr. Philip Felgner, University of California, Irvine. Plates were coated with MVA protein WR113/D8L, blocked with Casein/TBS, dried and stored at 4 °C until use. Samples were diluted 1:200 in blocking buffer (1% Casein in TBS supplemented with 10% *E. coli* lysate). After incubating in blocking buffer at room temperature (RT) for 30 minutes to block anti-*E. coli* reactivity, samples were added to the plate in duplicate, 50 uL/well. Plates were incubated for 45 minutes at RT before washing 6 times with PBS. Secondary antibody (goat anti-human IgG conjugated to Horseradish Peroxidase, ADI, H-HuG.211) was added 100 uL/well at a dilution of 1:100 in PBS and plates were incubated at RT for 45 minutes. Plates were washed 6 times in PBS before adding 100 uL of TMB substrate (ADI, 80091) per well and covering plates to protect from light. After 10 minutes, the reaction was stopped by adding 100 uL/well of stop solution (ADI, 80101). Optical density (OD) was read at 450 nm on an ELx800 microplate reader (Biotek) with Gen5 software (version 2.07).

### Statistical Analyses

Analyses were performed using GraphPad Prism, version 6 for Windows (GraphPad Software, La Jolla, California). Data tested negative for a Normal distribution by D’Agostina-Pearson omnibus normality test, therefore data are tested according to non-parametric tests and described using the median with interquartile range. Significance testing used the 2-tailed Mann–Whitney test for comparison of 2 groups, and the Kruskal–Wallis test with Dunn’s correction for comparisons between multiple groups. Wilcoxon matched pairs test and Friedman tests were used for paired analyses. Missing values for AUC analysis were generated through inferred values of group mean at missing time points. Volunteers were excluded if >3 time points missing. AUC was calculated per volunteer and group medians calculated. For AUC analyses of the 9 possible regimens, the 7 4-vaccination groups were compared against the prime-boost AM group only, which was the most immunogenic 2-vaccination regimen from ELISpot analysis. For ICS analyses of previous correlates of protection (CD8^+^ monofunctional IFN-γ^+^ and CD8^+^ CD107a^+^ [19]), regimens were compared against the A_M regimen the correlates were identified from. Correlations were assessed using the Spearman rank correlation coefficient with 2-tailed p-value. Safety profiles were assessed by Chi-Squared and Fisher’s exact analyses. The significance threshold was p-value <0.05.

## Electronic supplementary material

Supplementary tables and figures
