# Supplementary material for: Assessment of novel vaccination regimens using viral vectored liver stage malaria vaccines encoding ME-TRAP
Source: Sci Rep. 2018 Feb 21;8:3390. doi: 10.1038/s41598-018-21630-4 (PMC5821890; doi:10.1038/s41598-018-21630-4)

Assessment of novel vaccination regimens using viral vectored liver stage malaria vaccines encoding ME-TRAP.

Carly M Bliss*[1], Georgina Bowyer[1], Nicholas A Anagnostou[1], Tom Havelock[2], Claudia M Snudden[1], Huw Davies[3], Simone C de Cassan[1], Amy Grobbelaar[1], Alison M Lawrie[1], Navin Venkatraman[1], Ian D Poulton[1], Rachel Roberts[1], Pooja B Mange[1], Prateek Choudhary[1], Saul N Faust[2], Stefano Colloca[4], Sarah C Gilbert[1], Alfredo Nicosia[4,5,6], Adrian VS Hill[1], Katie J Ewer[1].

[1] The Jenner Institute, University of Oxford, Oxford, UK.

[2] NIHR Wellcome Trust Clinical Research Facility, University of Southampton, University Hospital Southampton NHS Foundation Trust, Southampton, UK.

[3] Department of Medicine, Division of Infectious Diseases, University of California, Irvine, CA, USA.

[4] ReiThera (formerly Okairos), 00144 Rome, Italy.

[5] CEINGE, Via Comunale Margherita, 484-538, 80131 Napoli, Italy.

[6] Department of Molecular Medicine and Medical Biotechnology, University of Naples Federico II, Naples, Italy

Correspondence should be addressed to:

Carly Bliss, Jenner Institute, Old Road Campus Research Building, Roosevelt Drive, Oxford, OX3 7DQ.

Email: carly.bliss@ndm.ox.ac.uk

Running Title: Novel vaccination regimens against liver stage malaria.

Supplementary Tables

Table S1a. Individual adverse events graded as severe after vaccination with ChAd63 ME-TRAP.

Volunteer	Duration (d)	Relatedness to vaccination	Adverse event	Study Group	Vaccine Number
10431111	0	Probable	Shivering	5	1
10431119	2	Definite	Vaccine Site Swelling	5	1
2043 1182	8	Possible	Headache	6	3
2043 1182	7	Possible	Sore throat	6	3
10431124	0	Possible	Nausea/Vomiting	6	1
10431124	0	Possible	Malaise	6	1

Table S1b. Individual adverse events graded as severe after vaccination with MVA ME-TRAP.

Volunteer	Duration (d)	Relatedness to vaccination	Adverse event	Study Group	Vaccine Number
MVT-10431105	4	Definite	Vaccine Site Pain	1	4
MVT-10431113	4	Definite	Vaccine Site Pain	3	3
MVT-10431113	0	Possible	Malaise	3	3
MVT 2043 1176	5	Definite	Vaccine Site Pain	4	2
MVT-10431114	2	Probable	Feverish	4	2
MVT-10431122	1	Probable	Arthralgia	4	4
MVT-10431122	1	Probable	Myalgia	4	4
MVT-10431119	4	Probable	Vaccine Site Erythema	5	2
MVT-10431119	2	Probable	Vaccine Site Swelling	5	2
MVT 2043 1182	2	Definite	Vaccine Site Swelling	6	2
MVT 2043 1182	3	Definite	Vaccine Site Pain	6	2
MVT 2043 1182	1	Definite	Malaise	6	2
MVT-10431135	0	Probable	Feverish	6	2
MVT-10431135	0	Probable	Malaise	6	2
MVT-10431135	0	Probable	Fatigue	6	2
MVT 2043 1173	7	Definite	Vaccine Site Swelling	7	2
MVT-10431137	2	Definite	Vaccine Site Pain	7	2
MVT-10431137	2	Probable	Feverish	7	2

Table S2. Number of local and systemic adverse events reported in each group after each vaccination, stratified according to severity. N/V, nausea and/or vomiting. N, number of volunteers at each vaccination.

Group	Vaccine	no. doses	n	Local Adverse Events								Systemic Adverse Events									
				Severity	Pain	Swelling	Erythema	Warmth	Scaling	Pruritus	Other	Fever	Feverish	Arthralgia	Myalgia	Malaise	Fatigue	Headache	N/V	Other	Laboratory
1	ChAd63	3	6	Mild	10	4	9	6	0	2	0	1	5	2	4	3	7	5	2	3	1
				Moderate	3	0	0	0	0	0	0	1	2	1	0	0	2	7	0	1	0
				Severe	0	0	0	0	0	0	0	0	0	0	0	0	0	0	0	0	0
	MVA	1	6	Mild	2	4	5	5	0	0	0	0	2	3	2	4	5	3	1	0	0
				Moderate	3	1	0	0	0	0	0	1	1	0	2	0	0	1	0	1	0
				Severe	1	0	0	0	0	0	0	0	0	0	0	0	0	0	0	0	0
2	ChAd63	2	6	Mild	4	3	5	1	0	1	0	0	5	2	2	4	6	5	2	0	0
				Moderate	0	0	0	0	0	0	0	1	0	1	1	1	0	1	1	0	0
				Severe	0	0	0	0	0	0	0	0	0	0	0	0	0	0	0	0	0
	MVA	2	6	Mild	7	7	9	8	0	3	0	0	1	3	4	6	3	4	1	3	0
				Moderate	4	1	1	1	0	0	0	0	0	1	1	0	1	0	0	1	0
				Severe	0	0	0	0	0	0	0	0	0	0	0	0	0	0	0	0	0
3	ChAd63	2	6	Mild	7	3	7	7	0	1	0	0	0	2	5	4	5	3	3	2	0
				Moderate	0	1	0	0	0	1	0	0	1	0	0	0	1	3	0	1	0
				Severe	0	0	0	0	0	0	0	0	0	0	0	0	0	0	0	0	0
	MVA	2	6	Mild	8	7	10	5	0	1	1	1	2	0	7	3	8	5	4	3	1
				Moderate	3	1	0	1	0	0	0	0	1	1	0	1	0	2	0	0	0
				Severe	1	0	0	0	0	0	0	0	0	0	0	1	0	0	0	0	0
4	ChAd63	1	6	Mild	5	0	4	4	1	1	0	0	2	3	4	0	3	3	1	2	0
				Moderate	0	1	0	0	0	0	0	0	0	0	0	0	0	0	0	0	0
				Severe	0	0	0	0	0	0	0	0	0	0	0	0	0	0	0	0	0
	MVA	3	6	Mild	12	13	18	13	0	2	2	3	7	6	7	5	10	3	1	1	0
				Moderate	5	2	0	0	0	0	0	0	0	0	0	0	2	1	0	1	0
				Severe	1	0	0	0	0	0	0	0	1	1	1	0	0	0	0	0	0
5	ChAd63	2	6	Mild	6	1	4	3	0	2	1	0	2	2	3	5	4	6	3	3	0
				Moderate	0	0	0	0	0	0	0	0	0	0	0	0	1	0	0	1	0
				Severe	0	1	0	0	0	0	0	0	0	0	0	0	0	0	0	1	0
	MVA	2	6	Mild	10	6	11	9	0	2	1	1	2	0	2	4	8	4	3	2	0
				Moderate	2	1	1	0	0	0	0	0	1	0	1	0	0	1	0	0	0
				Severe	0	1	0	0	0	0	0	0	0	0	0	0	0	0	0	0	0
6	ChAd63	2	6	Mild	7	2	9	4	0	0	1	0	1	1	4	3	4	3	1	3	0
				Moderate	1	0	0	0	0	0	0	0	1	0	1	0	0	2	0	0	0
				Severe	0	0	0	0	0	0	0	0	0	0	0	1	0	1	1	1	0
	MVA	2	6	Mild	3	8	12	8	0	0	0	0	1	1	3	1	3	2	0	3	0
				Moderate	8	3	0	0	0	0	0	0	0	0	3	2	2	4	0	0	0
				Severe	1	1	0	0	0	0	0	0	1	0	0	2	1	0	0	0	0
7	ChAd63	2	6	Mild	7	4	8	0	0	0	0	3	3	2	4	3	4	4	3	0	0
				Moderate	1	0	0	0	0	0	0	0	1	1	1	1	1	2	0	2	0
				Severe	0	0	0	0	0	0	0	0	0	0	0	0	0	0	0	0	0
	MVA	2	6	Mild	5	9	12	5	0	0	1	4	2	2	4	1	5	4	3	4	0
				Moderate	6	1	0	0	0	0	0	0	0	1	1	0	1	4	0	1	0
				Severe	1	1	0	0	0	0	0	0	1	0	0	0	0	0	0	0	0

Supplementary Figures

Figure S1. Sample gating strategy for multi-parameter flow cytometry with intracellular cytokine staining.

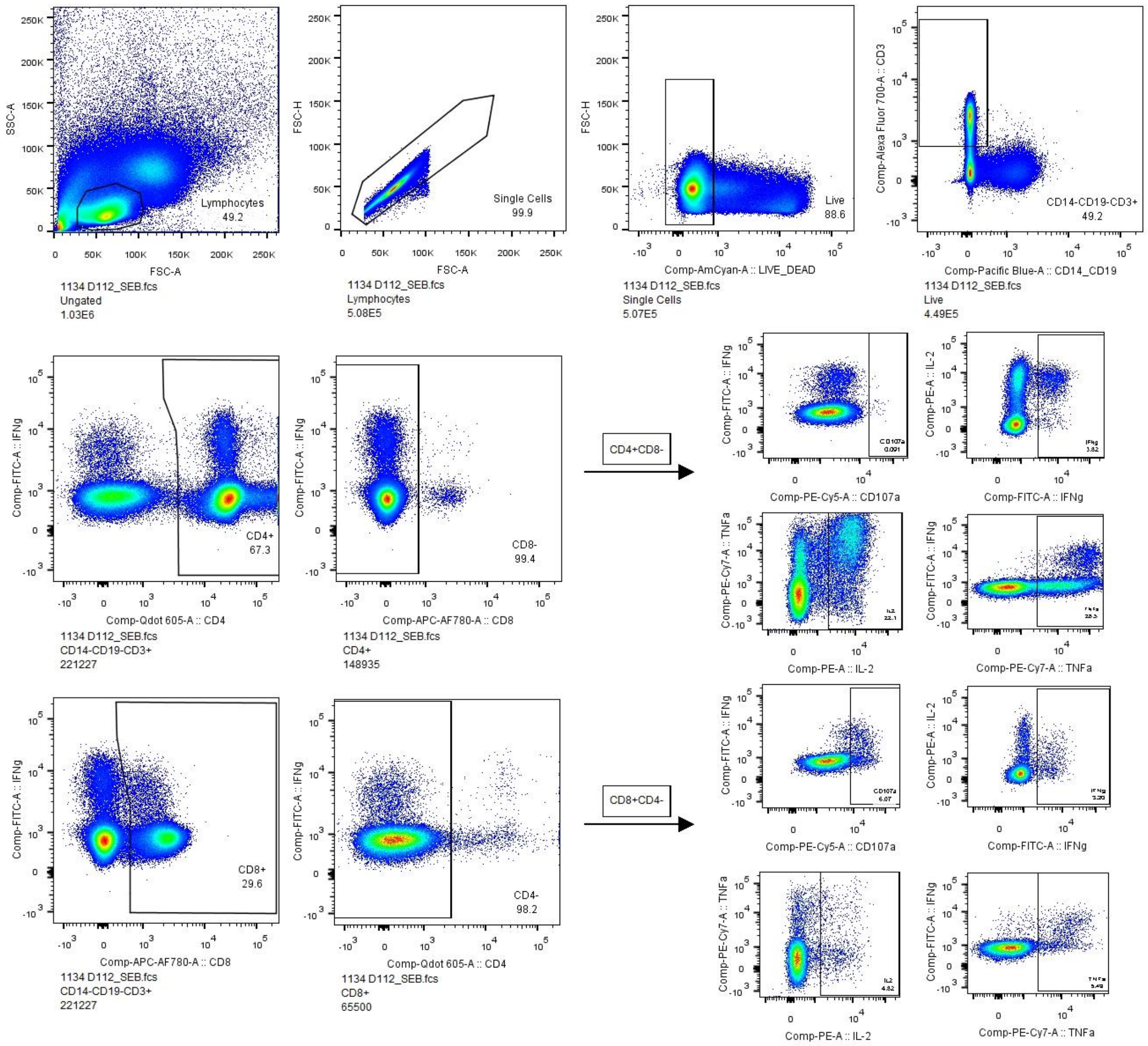

Figure S2. CONSORT diagram displaying volunteer enrolment, exclusion, allocation and follow up.

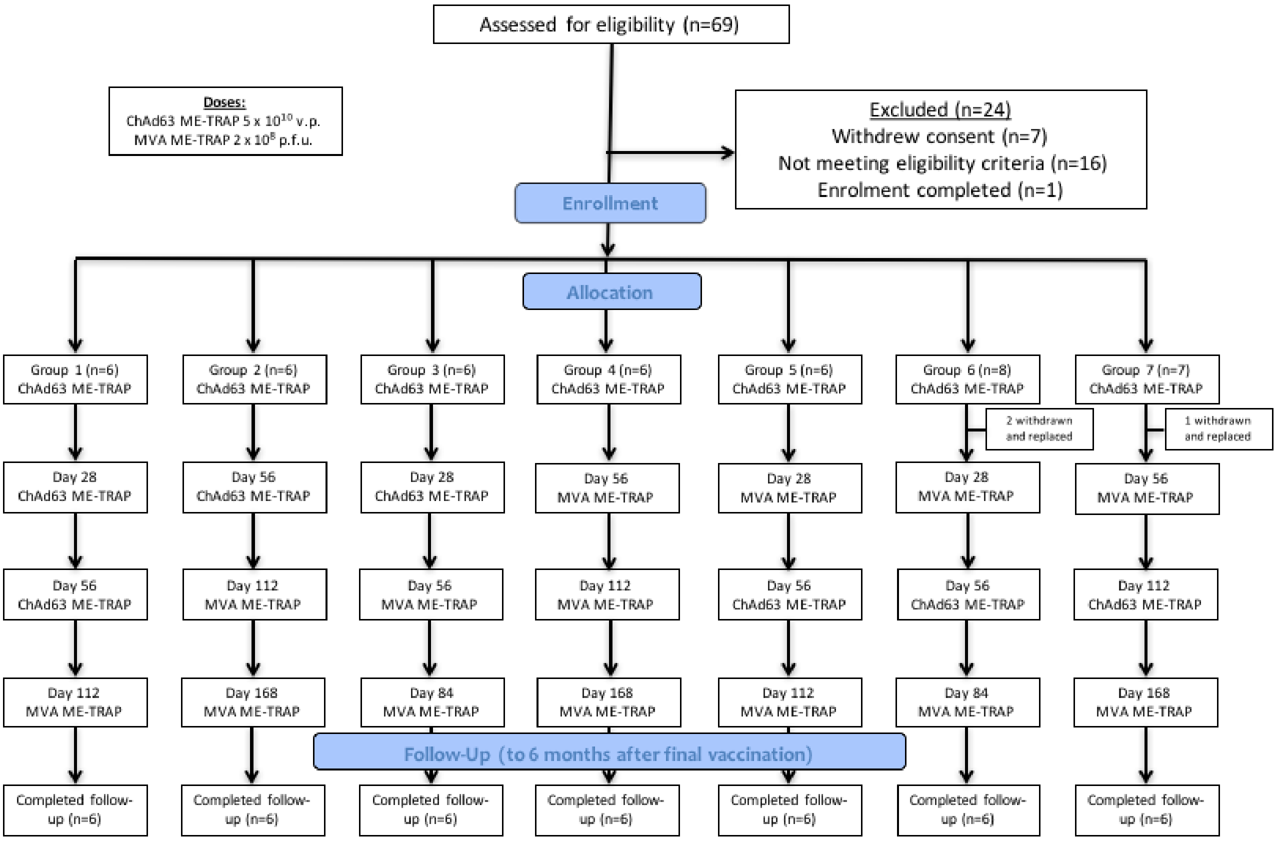

Supplement: Supplementary file 1 — Supplementary tables and figures [file 41598_2018_21630_MOESM1_ESM.pdf]
